# Budd-Chiari Syndrome Treated With IVC Stenting With Subsequent Right Hepatic Vein to IVC Retrograde Sharp Recanalization

**DOI:** 10.1155/crhe/2216461

**Published:** 2025-05-15

**Authors:** Andrew C. Gordon, Kush R. Desai, Justin R. Boike, Bartley G. Thornburg

**Affiliations:** ^1^Department of Radiology, Section of Interventional Radiology, Northwestern University, Chicago, Illinois, USA; ^2^Department of Medicine, Division of Gastroenterology and Hepatology, Northwestern University, Chicago, Illinois, USA

**Keywords:** hepatic vein, inferior vena cava, venous web

## Abstract

Venous webs of the inferior vena cava (IVC) and hepatic veins are rare and can result in Budd-Chiari syndrome. Included images of classic venous webbing are from a 60-year-old woman who presented with abdominal distension/pain, lower extremity edema, elevated liver tests, and ascites due to multifocal venous webbing of the IVC and right hepatic vein. The patient was successfully treated with IVC venous stent placement. Delayed recurrence of ascites, abdominal distention, and liver dysfunction after 3 years of follow-up was attributed to an additional right hepatic vein web/occlusion that was treated with a hepatic vein to IVC recanalization and stenting. The patient had complete resolution of symptoms at 2-year follow-up.

## 1. Introduction

Budd-Chiari syndrome (BCS) is uncommon with an estimated incidence of 1 in 100,000, and the majority of cases are due to thrombotic syndromes, making this report of nonthrombotic Budd-Chiari pathophysiology from venous webbing exceedingly rare [1]. Case reporting suggests systemic lupus erythematosus (SLE) may initially present with BCS due to antiphospholipid syndrome (APS) [2]. This report highlights successful long-term Interventional Radiology management.

## 2. Case Presentation

A 60-year-old woman with spina bifida, staghorn calculi, SLE, Sjögren disease, heart failure with preserved ejection fraction, and obstructive sleep apnea presented with diffuse abdominal pain, bilateral lower extremity edema, and high Serum-Ascites Albumin Gradient ascites and high total protein indicative of portal hypertension from a post hepatic etiology and elevated liver chemistries.

### 2.1. Diagnostic Workup

Postcontrast abdominal computed tomography (CT) was concerning for nonthrombotic BCS. The patient had an extensive rheumatologic workup that was positive for ANA (titer 1:1280) and a lupus anticoagulant in two instances more than 12 weeks apart suggestive of antiphospholipid antibody syndrome (APS); however, there was no prior history of thrombotic disorder or venous thromboembolism. After hepatology evaluation, the patient was referred for transjugular liver biopsy that was compatible with post-sinusoidal portal hypertension and a significant right atrium to IVC pressure gradient (16–20 mmHg). The patient was taken for venography and definitive treatment of ascites and symptoms of abdominal distension and lower extremity edema.

### 2.2. Management/Interventions

**Figure 1:**
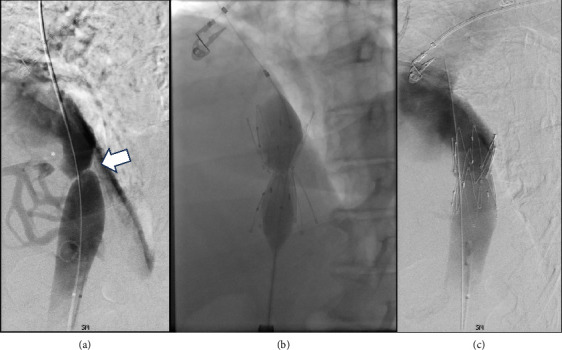
(a) Inferior vena cava venogram demonstrating a narrow web (denoted by the arrow) of the intrahepatic IVC with a translesional pressure gradient of 16 mmHg (above stenosis 8 mmHg, below stenosis 24 mmHg). Multiple hepatic venous collaterals also highlight a potential web/occlusion of the right hepatic vein (denoted by the asterisk). (b) A 30 × 50 mm Gianturco Z stent (Cook Medical, Bloomington, IN) was successfully deployed across the stenosis and subsequently postdilated. (c) Completion venography and intravascular ultrasound demonstrate subtotal luminal restoration with a decreased pressure gradient of 1 mmHg (above prior stenosis 18 mmHg, below prior stenosis 19 mmHg).

Cavography demonstrated a web of the intrahepatic IVC (Figure 1) with a significant translesional pressure gradient of 16 mmHg. Intravascular ultrasound demonstrated > 90% stenosis of the intrahepatic IVC. The stenosis was treated with placement of a 30 mm Gianturco Z-stent (Cook Medical, Bloomington, IN), decreasing the gradient to 1 mmHg. She was discharged on diuretics with complete resolution of ascites and associated symptoms including abdominal distension and lower extremity edema with normalization of liver chemistries. Diuretics were discontinued shortly thereafter.

### 2.3. Follow-Up

**Figure 2:**
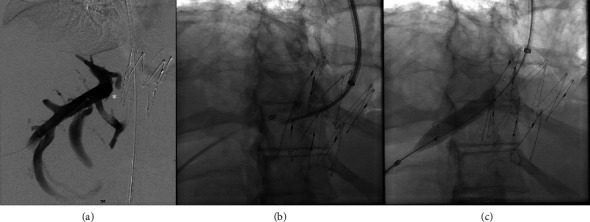
(a) Venography through 5Fr Accustick demonstrated multiple prominent intrahepatic venovenous collaterals to segment-4, sluggish flow with to-and-fro motion in the right hepatic vein and an abrupt occlusion/cutoff of the central right hepatic vein (asterisk), near the right posterior aspect of the existing Gianturco Z-stent. There was a significant translesional pressure gradient of 13 mmHg (IVC 7 mmHg, right hepatic vein 20 mmHg). (b) Attempts to cross this lesion antegrade including sharp recanalization technique were not successful and the decision was made to cross retrograde with a Colapinto needle. (c) A 12 × 40 mm S.M.A.R.T. stent (Cordis, Santa Clara, CA) crossing the lesion and extending through the interstices of the existing Gianturco Z-stent was deployed in the IVC and postdilated with a 12 mm balloon in multiple stations with a narrow waist at the occlusion/lesion that eventually resolved.

**Figure 3:**
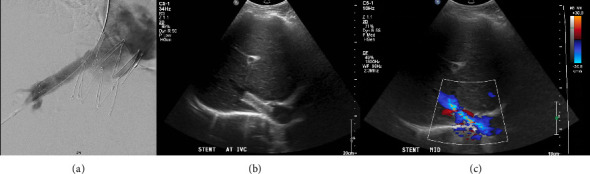
(a) Venography demonstrated patency of the newly placed stent with brisk antegrade flow into the IVC and no significant filling of the intrahepatic venovenous collaterals with reduction of the translesional gradient to 1 mmHg (IVC 15 mmHg, right hepatic vein 16 mmHg). (b, c) Grayscale and color Doppler liver ultrasound 10 months after the procedure demonstrated no ascites and patency of the hepatic venous stent. At 2-year follow-up, the patient had no recurrence of ascites or liver dysfunction.

After 3 years, there was a gradual recurrence of ascites and abdominal distension. Repeat CT demonstrated IVC stent patency; however, there was segmental mottling of the liver parenchyma in right hepatic lobe. The right hepatic vein could not be catheterized from a transjugular approach, and therefore direct transhepatic venography was performed demonstrating webbing/occlusion of the right hepatic vein with multiple venovenous collaterals draining into segment 4 (Figure 2). Using the transhepatic access, multiple attempts to cross the fibrotic occlusion, including sharp techniques, were not successful. The decision was made to sharply recanalize the occlusion via a transjugular approach with a puncture from the IVC to the right hepatic vein with a Colapinto needle (Cook Medical, Bloomington, IN) to recanalize the hepatic vein and IVC; a snare was placed as a landmark in the right hepatic vein via transhepatic access. A 12 × 40 mm stent (SMART Control, Cordis, Santa Clara, CA) was placed across the lesion, extending through the interstices of the existing Gianturco Z-stent in the IVC. Completion venography demonstrated patency of the newly placed stent with brisk antegrade flow into the IVC and no significant filling of the intrahepatic venovenous collaterals (Figure 3). No antithrombotic therapy was required after either intervention. On follow-up ultrasound at 10 months, venous stents were demonstrated to be patent; with complete resolution of ascites and associated abdominal distension through 24 months.

## 3. Discussion

The incidence of inferior vena cava web is unknown; however, these lesions are rare and theorized to result from either a congenital/developmental vascular anomaly or related to a fibrotic band/membrane formation due to prior thrombosis [3–5]. The current case demonstrates that venous webs can be multifocal, can result in both liver and lower extremity symptoms, and can be readily managed with transcatheter techniques. Prior reported treatments have included venoplasty, stent placement, and transjugular intrahepatic portosystemic shunt (TIPS) [4, 5]. In this case, symptomatic BCS resulted from hepatic venous outflow obstruction with postsinusoidal portal hypertension, and symptomatically manifested with ascites, lower extremity edema, and elevated liver chemistries. Despite successful initial treatment with IVC stent placement, an additional web/occlusion of the right hepatic vein was only noted in retrospect after 3 years upon recurrence of ascites. The pathophysiology behind why it took 3 years for hepatic dysfunction and ascites to recur is not clear but one theory would be relative hepatic recovery and compensation overall from relieved hepatic congestion with a smaller sector of liver with continued worsening congestion and compensation via the venovenous collaterals to segment 4. Therefore, the initial diagnosis and treatment with improvement of symptoms yielded no clinical indication for hepatic venous stenting until recurrence of symptoms 3 years later. Our preferred technical approach for crossing the hepatic venous lesion would have been from antegrade transhepatic access as this would have not required transjugular access or sharp recanalization; however, the lesion could not be crossed with conventional or sharp techniques. Alternatives discussed for recanalization included a radiofrequency wire (Powerwire, Baylis MedTech, Montreal, Canada), gun-sight technique, and sharp retrograde crossing with a TIPS needle. To avoid additional hepatic punctures or the creation of a new transhepatic tract, ultimately, a readily available TIPS needle was utilized with successful traversal and stent placement for an anatomic hepatic vein to IVC retrograde recanalization in contrast to prior antegrade transhepatic techniques reported. Nonanatomic right hepatic vein to middle hepatic vein approaches for IVC recanalization and membrane balloon venoplasty have also been described [6, 7]. In this case, the chronicity and fibrotic nature of the occlusion and location abutting an IVC stent warranted stent placement.

## 4. Conclusions

This case of nonthrombotic BCS in a SLE patient highlights that venous webbing is often multifocal with the potential for severe and recurrent symptoms resulting from venous obstruction. IVC stent placement and a hepatic vein to IVC retrograde recanalization with stent placement resulted in the complete resolution of symptoms.

## Data Availability

Data are available from the authors upon reasonable request.

## References

[1] Ferral H., Behrens G., Lopera J. (2012). Budd-Chiari Syndrome. *American Journal of Roentgenology*.

[2] Solela G., Daba M. (2023). Budd-Chiari Syndrome as an Initial Presentation of Systemic Lupus Erythematosus Associated With Antiphospholipid Syndrome: A Case Report with Review of the Literature. *Open Access Rheumatology Research and Reviews*.

[3] Smillie R. P., Shetty M., Boyer A. C., Madrazo B., Jafri S. Z. (2015). Imaging Evaluation of the Inferior Vena Cava. *RadioGraphics*.

[4] Strozzi M., Besic K. M., Ivana K. S., Darko A. (2020). Endovascular Treatment of an Obstructive Membrane between Inferior Vena Cava and Right Atrium in an Unrecognized Budd-Chiari Syndrome. *CVIR Endovasc*.

[5] Bozorgmanesh A., Selvam D. A., Caridi J. G. (2007). Budd-Chiari Syndrome: Hepatic Venous Web Outflow Obstruction Treated by Percutaneous Placement of Hepatic Vein Stent. *Seminars in Interventional Radiology*.

[6] Sujanyal S. A., Shah P. P., Willis J. G., El Khudari H., Varma R. K. (2023). Transhepatic Inferior Vena Cava Recanalization in a Case of Budd Chiari Syndrome: A Novel Approach. *Radiology Case Reports*.

[7] Metzger P. B., Costa K. R., Silva S. L. E. (2021). Budd-Chiari Syndrome Due to Hepatic Venous Web Outflow Obstruction: Percutaneous Treatment with Balloon Angioplasty. *Jornal Vascular Brasileiro*.

